# UK guidelines for the management of soft tissue sarcomas

**DOI:** 10.1186/s13569-016-0060-4

**Published:** 2016-11-15

**Authors:** Adam Dangoor, Beatrice Seddon, Craig Gerrand, Robert Grimer, Jeremy Whelan, Ian Judson

**Affiliations:** 1 Bristol Cancer Institute, Bristol Haematology & Oncology Centre, University Hospitals Bristol NHS Trust, Bristol, BS2 8ED UK; 2 Department of Oncology, University College London Hospital NHS Trust, London, NW1 2PG UK; 3 The Newcastle upon Tyne Hospitals NHS Foundation Trust, Freeman Hospital, Newcastle-upon-Tyne, NE7 7DN UK; 4 Royal Orthopaedic Hospital NHS Trust, Birmingham, B31 2AP UK; 5 Royal Marsden NHS Foundation Trust, London, SW3 6JJ UK

## Abstract

Soft tissue sarcomas (STS) are rare tumours arising in mesenchymal tissues, and can occur almost anywhere in the body. Their rarity, and the heterogeneity of subtype and location means that developing evidence-based guidelines is complicated by the limitations of the data available. However, this makes it more important that STS are managed by teams, expert in such cases, to ensure consistent and optimal treatment, as well as recruitment to clinical trials, and the ongoing accumulation of further data and knowledge. The development of appropriate guidance, by an experienced panel referring to the evidence available, is therefore a useful foundation on which to build progress in the field. These guidelines are an update of the previous version published in 2010 (Grimer et al. in Sarcoma 2010:506182, 2010). The original guidelines were drawn up following a consensus meeting of UK sarcoma specialists convened under the auspices of the British Sarcoma Group (BSG) and were intended to provide a framework for the multidisciplinary care of patients with soft tissue sarcomas. This current version has been updated and amended with reference to other European and US guidance. There are specific recommendations for the management of selected subtypes of disease including retroperitoneal and uterine sarcomas, as well as aggressive fibromatosis (desmoid tumours) and other borderline tumours commonly managed by sarcoma services. An important aim in sarcoma management is early diagnosis and prompt referral. In the UK, any patient with a suspected soft tissue sarcoma should be referred to one of the specialist regional soft tissues sarcoma services, to be managed by a specialist sarcoma multidisciplinary team. Once the diagnosis has been confirmed using appropriate imaging, plus a biopsy, the main modality of management is usually surgical excision performed by a specialist surgeon. In tumours at higher risk of recurrence or metastasis pre- or post-operative radiotherapy should be considered. Systemic anti-cancer therapy (SACT) may be utilized in some cases where the histological subtype is considered more sensitive to systemic treatment. Regular follow-up is recommended to assess local control, development of metastatic disease, and any late-effects of treatment. For local recurrence, and more rarely in selected cases of metastatic disease, surgical resection would be considered. Treatment for metastases may include radiotherapy, or systemic therapy guided by the sarcoma subtype. In some cases, symptom control and palliative care support alone will be appropriate.

## Background

### Rationale and objective of guidelines

Soft tissue sarcomas (STS) are a relatively uncommon group of malignancies. On average a general practitioner may only see one sarcoma in their career. To improve diagnosis and treatment of these tumours, management was rationalized to peer-reviewed regional soft-tissue sarcoma services, and a smaller number of specialist units which also treat primary bone tumours [1]. An outline of best practice was set out in the National Institute for Health and Clinical Excellence Improving Outcomes Guidance for people with sarcoma (NICE-IOG) [2] published in 2006.

These guidelines are an attempt to review current evidence concerning soft-tissue sarcoma diagnosis and treatment, and provide recommendations to support best practice. They are not intended to be prescriptive, but aim to improve the quality of care for patients with STS by helping identify and inform the key decisions involved in their management. They will hopefully provide a useful resource for sarcoma services to help guide multidisciplinary team (MDT) case discussions, and patient management.

### Methods

This updated guideline has been authored and reviewed by specialists from the UK involved in diagnosing and treating patients with sarcoma. They include members of the British Sarcoma Group (BSG), and NHS England Sarcoma Clinical Reference Group (CRG). As with the previous version, current NICE, NCCN (National Comprehensive Cancer Network, US), and ESMO (European Society for Medical Oncology) guidance were referenced, tailoring the recommendations for UK practice. It provides a brief review of the current state of established knowledge in sarcoma diagnosis and management, with guidance on what is considered current best practice in the UK. It has been derived by a consensus of expert opinion based on their interpretation of currently available data, and their own clinical experience.

### Scope of guidelines

These recommendations apply principally to soft tissue sarcomas arising from limbs and trunk and although, where appropriate, specific guidance is given according to histological subtype it is recognised that some tumours, for example, Ewing sarcoma, and embryonal and alveolar rhabdomyosarcoma, require a different approach to management, and are excluded from this guidance [3]. These rare subtypes are relatively more common in paediatric and young adult patients. Ewing sarcoma arising in soft tissue are managed in accordance with guidelines for Ewing sarcoma of bone (see UK bone sarcoma guidelines [4]). Rhabdomyosarcoma is the commonest sarcoma in children and appropriately managed by children’s cancer multidisciplinary teams (MDTs), often within international clinical trials such as EpSSG RMS 2005 [5, 6] which include comprehensive treatment guidance. For other histologies arising in children and young people (often referred to as non-rhabdomyosarcoma, soft tissue sarcomas, NRSTS) much less evidence exists for optimal management, in particular the application of chemotherapy and radiotherapy. Close working between children’s cancer MDTs and sarcoma MDTs should be regarded as best practice.

Specific recommendations on the management of retroperitoneal and uterine sarcomas, as well as aggressive fibromatosis (desmoid tumours), plus some other conditions referred commonly to sarcoma MDTs, are included separately within this guideline. Bone sarcomas and gastrointestinal stromal tumours (GISTs) are subject to their own specific BSG guidelines. The latest bone sarcoma guidelines have recently been published [4] and the GIST guidelines are currently being updated.

These guidelines focus on clinical effectiveness, giving a picture of what treatments a specialist sarcoma multidisciplinary team should have access to within the UK, subject to some flexibility to allow for evolving practice, but they do not employ the same detailed analysis of cost effectiveness as NICE. In rare situations, treatment options may be suggested where NHS funding is not established. Unfortunately, with rare tumours such as sarcoma, NICE, and the Cancer Drugs Fund (CDF), may be less likely to evaluate potential treatments. These guidelines can be considered to represent a broad consensus in 2016. They will require updating as knowledge and treatment evolve.

### Specialised soft-tissue sarcoma services

Following the publication of the National Institute for Health and Clinical Excellence Improving Outcomes Guidance for people with sarcoma (NICE-IOG) [2] in 2006, the services for patients with sarcoma in England and Wales were centralised. There are currently five centres providing both bone and soft-tissue sarcoma services, and an additional ten centres who diagnose and treat only soft-tissue sarcoma, passing on referrals of suspected bone sarcomas to their regional bone sarcoma centre [7] and collaborating on aspects of management. In England, services are commissioned and delivered in accordance with the current NHS England service specification [8]. In Scotland, the Scottish Sarcoma Network coordinates care of patients at five centres, and in Northern Ireland all bone sarcomas are managed at Musgrave Park Hospital with soft-tissue sarcomas also seen at four other centres.

Each specialist service must have a multidisciplinary team (MDT) made up of radiologists, surgeons, medical and clinical oncologists, pathologists, specialist nurses, and an MDT co-ordinator. The surgical team will include specialist plastic, general, or orthopaedic surgeons, with an extended team available, which may include retroperitoneal, thoracic, vascular, and other surgical disciplines, plus allied health professionals such as physiotherapists and occupational therapists. Supportive and palliative care services also contribute. The MDT will hold weekly meetings to discuss new suspected, and proven cases of sarcoma. The MDT meeting outcomes should be provided promptly to referring clinicians.

## Epidemiology

Sarcomas are relatively uncommon tumours accounting for approximately 1% of all adult cancers [9]. They constitute a heterogeneous group of tumours of mesenchymal cell origin, often with a distinct age distribution, site of presentation, natural biological behaviour and prognosis. There are more than 50 subtypes divided into two broad categories: soft tissue sarcomas and sarcomas of bone [10].

Historically, because of the heterogeneity of this group of tumours, the true incidence has generally been under-reported. In 2010 around 3300 people were diagnosed with soft tissue sarcoma in the UK, with around 90 cases in children under 15. In the Teenage and Young Adult (TYA) age range (17–25 years) around 80 cases were recorded [11]. The National Cancer Intelligence Network (NCIN) reports that the incidence of STS is approximately 45/million population per year (NCIN) [12]. Bone sarcomas are rarer with an incidence around a fifth that of STS; 559 new cases were recorded in 2011 [11]. However, they represent a significant proportion of the cancer burden in young people under the age of 20 years.

Soft tissue sarcomas may occur at any age, most often in middle aged and older adults; however, as a proportion of paediatric malignancies they are relatively common comprising 7–10% of all childhood cancers. They are an important cause of death in the 14–29 years’ age group [13–16].

Approximately half of all STS patients with intermediate or high-grade tumours develop metastatic disease requiring systemic treatment [17]; the overall survival is approximately 55% at 5 years [12, 18].

## Aetiology

For the vast majority of cases, the aetiology is unknown, although there are certain genetic associations, such as the 10% lifetime risk of malignant peripheral nerve sheath tumour (MPNST) in individuals with familial neurofibromatosis, caused by mutations in the *NF1* gene [19, 20]. There is an increased risk of sarcomas, both bone and soft tissue, in patients who have had a familial retinoblastoma, caused by inherited mutations in the *RB* gene [21]. Similarly, there is an increased risk of sarcomas, and other cancers in families with Li-Fraumeni syndrome who have inherited mutations in the *TP53* tumour suppressor gene [22]. There is also a small risk of sarcoma in areas of the body previously treated using radiotherapy, for example angiosarcoma following treatment for breast cancer.

## Clinical presentation

Due to the heterogeneous sites of origin of STS, it is difficult to clearly define the clinical features of the disease. However, a soft tissue lump exhibiting any of the following three clinical features should be considered to be malignant until proved otherwise [23]:

1. Increasing in size.
2. Size more than 5 cm.
3. Painful.

The more of these clinical features present, the greater the risk of malignancy with increasing size being the best individual indicator. In addition, deeper lying masses are more likely to be sarcomas.

Soft tissue masses are not uncommon and most will turn out to be benign, often lipomata. NICE produced updated guidelines in 2015, aimed at primary care, for early diagnosis of soft tissue sarcomas [24]. They suggest that the criteria for urgent referral should be adhered to even if the risk of malignancy is only 3%.

- Consider an urgent direct access ultrasound scan (to be performed within 2 weeks) to assess for soft tissue sarcoma in adults with an unexplained lump that is increasing in size.
- Consider a suspected cancer pathway referral (for an appointment within 2 weeks) for adults if they have ultrasound findings that are suggestive of soft tissue sarcoma or if ultrasound findings are uncertain and clinical concern persists.

If there is a particularly high suspicion of malignancy, and requesting an ultrasound in the primary care setting might introduce delay, then direct urgent referral to the regional sarcoma service should be considered. Regional services should provide referral advice on their websites or urgent referral forms. Any lesions previously thought to be benign that increase in size or develop other suspicious features should be considered for further investigation. Other diagnoses to consider in the case of palpable masses include metastases and lymphoma.

Any retroperitoneal or intra-abdominal mass with imaging appearances suggestive of a soft tissue sarcoma should be referred to a specialist centre before biopsy or surgical treatment.

### Key recommendations

1. Any patient with a soft tissue mass that is increasing in size, or has a size more than 5 cm, whether or not it is painful, should either be referred for an urgent ultrasound scan, or referred directly to a sarcoma diagnostic centre.
2. If the ultrasound scan does not confidently confirm a benign diagnosis, then the patient should be referred for further investigation on an urgent suspected cancer pathway referral.
3. Any retroperitoneal or intra-abdominal mass with imaging appearances suggestive of a soft tissue sarcoma should be referred to a specialist centre before biopsy or surgical treatment.

## Referral and assessment

### Regional diagnostic services

The regional sarcoma services should support the development of efficient pathways for the investigation of suspected sarcomas. This may include providing information to local primary care or radiology services on the initial investigation and onward referral of patients with soft-tissue masses, and effective pathways to make direct suspected-cancer referrals when required.

Carcinosarcomas are generally viewed as epithelial tumours exhibiting sarcomatous differentiation. Although new biological insights are emerging [25], currently management would usually be as for epithelial tumours, and guided by the relevant cancer MDT.

### Imaging

#### Diagnostic

Any patient with a suspected STS should be referred for an initial ultrasound scan, or direct to a diagnostic centre for triple assessment with clinical history, imaging, and biopsy [24]. An initial ultrasound is often useful in lower-risk cases to confirm benign conditions such as simple lipomata. In the hands of a non musculo-skeletal ultrasonographer however errors may arise and so there should be a low threshold for referral for further investigation. A more definitive ultrasound may be performed by a musculoskeletal radiologist who ideally is a member of the sarcoma MDT. Any patients with suspicious ultrasound or clinical features should usually have an MRI scan of the region affected. Plain X-ray may be used to identify bone involvement and risk of fracture, or to detect calcification. For retroperitoneal tumours CT is often more convenient, and as useful as MRI.

#### Staging

Patients with a confirmed STS should be staged with a CT chest to exclude pulmonary metastases prior to definitive treatment, although plain chest X-ray may be acceptable in a minority of cases (e.g. the frail elderly and those with small, low grade lesions). In most cases CT abdomen/pelvis and isotope bone scan are not routine staging investigations, but CT may be considered, particularly in lower extremity tumours [26]. Depending on the histological type and other clinical features [26], further staging assessments may be advised as below:

- CT or MRI scan for regional lymph node assessment for synovial sarcoma, clear cell sarcoma, or epithelioid sarcoma due to a higher risk of nodal involvement.
- Atypical lipomatous tumours (ALT) of the extremities have a very low risk of metastatic spread and so chest X-ray may be considered adequate staging (see “Lipomas and atypical lipomatous tumours” section).
- In cases of myxoid liposarcoma soft-tissue metastases are more common and so abdominal and pelvic CT scan should be performed. Alternatively, although not yet established as routine practice, whole-body MRI has been shown to have potential utility in identifying occult metastatic disease and can be considered [27].
- Brain CT or MRI can be considered in cases of alveolar soft part sarcoma and clear cell sarcoma due to a higher incidence of brain metastases [28].
- Positron emission tomography (PET-CT) scanning is not yet proven as a routine investigation in sarcoma although may be considered before performing radical surgery, such as amputation for primary or recurrent disease [29]. It also provides a single investigation which can replace a separate CT and bone scan, and is being applied more commonly in sarcomas of younger patients such as Ewing sarcoma and rhabdomyosarcoma [30, 31]. Although some work has been done assessing tumour response to systemic treatment using PET; this is currently still investigational. PET-CT might have some utility in diagnosing neurofibromatosis 1 (NF1) associated malignant peripheral nerve sheath tumours (MPNST) [32, 33].

### Biopsy

The standard approach to diagnosis of a suspicious mass is percutaneous core needle biopsy—several cores should be taken to maximise diagnostic yield. However, an incisional biopsy may be necessary on rare occasions, and excision biopsy may be the most practical option for small superficial lesions (<2 cm diameter). Biopsies of large lipomatous lesions with concerning features, should aim to sample areas appearing more heterogeneous on imaging, and need to be interpreted with caution as areas of dedifferentiation may be missed. The biopsy should be planned in such a way that the biopsy tract can be safely removed at the time of definitive surgery to reduce the risk of seeding, and should be performed at a diagnostic clinic by, or in conjunction with, a specialist radiologist or sarcoma surgeon. Fine needle aspiration (FNA) is not recommended as a primary diagnostic modality, although it may be considered for confirming disease recurrence, or nodal metastases.

### Histology—diagnosis

Histological diagnosis should be made according to the 2013 WHO Classification [10] to determine the grade and stage of the tumour. The grade should be provided in all cases where possible based on a recognised system. In Europe, the Fédération Nationale des Centres de Lutte Contre le Cancer (FNCLCC) grading system is generally used, which distinguishes three grades (Table 1) [34, 35]. The mitotic rate should be recorded. Because of tumour heterogeneity, a core biopsy may not provide accurate information about grade [36]. In addition, certain translocation-driven sarcomas have a relatively uniform cellular morphology and, as such, can be misleadingly scored as intermediate, rather than high grade. This is especially true for myxoid/round cell liposarcomas, for which a different grading system based on the percentage of round cells is often used. Additional information may be provided by radiological imaging, and histology may be modified following assessment of the complete surgical resection specimen.

**Table 1:** FNCLCC histological grading criteria [34, 35]

Tumour differentiation	Necrosis	Mitotic count (n per 10 high power fields)
1. Well	0: Absent	1: n < 10
2. Moderate	1: <50%	2: 10–19
3. Poor (anaplastic)	2: ≥50%	3: n ≥ 20

The sum of the scores of the three criteria determines the grade of malignancy. Grade 1 = 2 or 3; Grade 2 = 4 or 5; Grade 3 = 6

Pathologic diagnosis relies on morphology and immunohistochemistry. Increasingly it should be complemented by molecular pathology to confirm those diagnoses characterised by a specific genetic abnormality, such as an activating mutation, chromosomal translocation, or chromosomal amplification, using for example fluorescent in situ hybridisation (FISH), or reverse transcription polymerase chain reaction (RT-PCR) [37]. It may have particular utility when the clinical pathologic presentation is unusual, or the histological diagnosis is doubtful. Molecular testing is now routine to confirm diagnoses such as Ewing sarcoma, rhabdomyosarcoma, synovial sarcoma, and to differentiate lipomas from atypical lipomatous tumours/well-differentiated liposarcomas.

#### Histology—resection

The report on the resected specimen should comply with the recommendations for reporting of STS produced by the Royal College of Pathologists [37]. The pathology report should include an appropriate description of tumour depth (in relation to the superficial fascia) and margins (whether they are intralesional, marginal, or wide, and include distance from surrounding tissues, or the presence of an anatomical barrier). The pathologic assessment of margins should be made in collaboration with the surgeon, and confirmation obtained as to whether the tumour was excised intact. Tumour size and grade should be documented noting that the latter cannot be reliably assessed after pre-operative treatment with radiotherapy or systemic therapy. In this setting the tumour may be assessed for histological response to treatment although the prognostic implications are not well established, in contrast to their utility in osteosarcoma or Ewing sarcoma of bone.

If feasible, it is recommended that tumour samples should be collected and frozen, both for future research and because new molecular pathological assessment techniques may become available later that could yield new information of direct value to the individual patient. Any tissue thus obtained is governed by the Human Tissue Authority; hence appropriate informed consent will need to be obtained from the patient.

#### Classification of margins

Historically, four categories of surgical margin have been described histologically: intralesional, marginal, wide and radical [38]. Whilst still included in the most recent ESMO guidance, they are summarised below.

##### Intralesional

Margin runs through tumour and therefore tumour remains.

##### Marginal

Surgical plane runs through pseudocapsule (reactive zone). The local recurrence rate is high because of tumour satellites in the reactive tissue. There are however prognostic differences between a planned and unplanned marginal excision.

##### Wide

Surgical plane is in normal tissue but in the same compartment as the tumour. The recurrence rate is low and is related only to skip lesions in the affected compartment.

##### Radical

The tumour is removed including affected compartments and there is a minimal risk of local recurrence.

However, the recent dataset from the Royal College of Pathologists [37] focuses more simply on the clearance in millimetres of the closest surgical margin, the type of tissue at the margin (e.g. fascia, fat, muscle or skin), whether the invasive margin is infiltrative or pushing, and presence or otherwise of vascular invasion. It is recognised that there is likely to be wide variation in the use of these descriptions and a more pragmatic approach, used in other cancer types, may be to simply classify the margins according to whether there is tumour at the cut edge or not:

R0—no tumour at the cut edge.

R1—tumour extends to cut edge.

R2—macroscopic residual tumour.

Margin assessment is complex and must take into account both the histological subtype of the resected sarcoma and the nature of the R1 resection margin. A positive resection margin at an intentionally preserved critical structure (planned margin) may have quite different prognostic significance to a multifocal R1 margin on the muscular surface of a resected specimen [39].

### Staging

The most commonly used staging system for soft-tissue sarcoma, produced by the American Joint Committee on Cancer [40], includes information on both the grade (Table 1) and stage of the tumour (Table 2). The 8th edition of the staging system will be published shortly and include consideration of anatomical location.

**Table 2:** AJCC TNM Classification for STS [40]

Classification	Description
Primary tumour (T)	
TX	Primary tumour cannot be assessed
T0	No evidence of primary tumour
T1	Tumour ≤5 cm in greatest dimension
	T1a Superficial tumour
	T1b Deep tumour
T2	Tumour >5 cm in greatest dimension
	T2a Superficial tumour
	T2b Deep tumour
Regional lymph nodes (N)	
NX	Regional lymph nodes cannot be assessed
N0	No regional lymph node metastasis
N1	Regional lymph node metastasis
Distant metastasis (M)	
M0	No distant metastasis
M1	Distant metastasis
Histologic grade (G)	
GX	Grade cannot be assessed
G1	Well-differentiated
G2	Moderately differentiated
G3	Poorly differentiated

Physical examination, diagnostic radiology and biopsy provide the AJCC criteria input data needed to stage STS

The final stage groupings are not altered whether the tumour is superficial or deep, and are thus as follows:

- Stage I
  - IA = low grade, small (G1/X, T1a/b, N0, M0).
  - IB = low grade, large (G1/X, T2a/b, N0, M0).
- Stage II
  - IIA = intermediate or high grade, small (G2/3, T1a/b, N0, M0).
  - IIB = intermediate grade, large (G2, T2a/b, N0, M0).
- Stage III
  - High grade, large, (G3, T2a/b, N0, M0).
  - Regional node involvement, with any size and grade of primary tumour (G1-3, T1-2, N1, M0).
- Stage IV
  - Metastasis identified (G1-3, T1-2, N0-1, M1).

### Key recommendations

1. All patients with a suspected STS should be managed by a specialist Sarcoma MDT as specified in the NICE guidance.
2. Ultrasound scan by a musculoskeletal radiologist should be considered as the first-line investigation, and may be supplemented by ultrasound-guided core biopsy.
3. Magnetic resonance imaging and core needle biopsy are recommended prior to definitive surgery.
4. Imaging of the thorax by CT scan for lung metastases should be done prior to radical treatment. Further staging may be considered depending on subtype and location of the sarcoma.

## Management of localised disease

Soft tissue sarcomas are a diverse group of tumours and, as our understanding of the differing natural history and response to treatment improves, it is increasingly possible to tailor treatment according to the individual histology. The major therapeutic goals are long-term survival, avoidance of local recurrence, maximising function, and minimising morbidity.

All patients should have their care managed by a formally constituted sarcoma MDT. Decisions about surgery, chemotherapy, radiotherapy and the timing of all these modalities should be made by the Sarcoma MDT. For site specific STS (e.g. gynaecological, head and neck) there should be a formal relationship between the sarcoma MDT and the site-specific MDT. In organising services in England reference should be made to the NICE quality standard for sarcoma [41] and the current NHS England sarcoma Service Specification [8]. The devolved nations may use these documents as reference but will have their own recommendations. Coordination with the sarcoma MDT helps to ensure optimal management of the sarcoma subtypes, recruitment to clinical trials, and enhances accurate data collection on sarcoma diagnoses and outcomes. In all cases the treatment options will be discussed with the patient, who should be supported by a specialist nurse.

For most limb and truncal tumours conservative surgery, in selected cases, combined with pre- or post-operative radiotherapy is standard treatment, and achieves high rates of local control whilst maintaining optimal function. Radiotherapy may be avoided in patients with low-grade tumours that have been completely resected, or those with small, superficial high-grade tumours resected with wide margins.

### Surgery

#### Surgery for localised disease

Surgery is the standard treatment for all patients with adult-type, localised soft tissue sarcomas, and should be performed by a surgeon who has appropriate training in the treatment of sarcoma. Evaluation of the resectability of a tumour is determined by the surgeon in consultation with the Sarcoma MDT, and depends on the tumour stage, the anatomical location, and the patient’s comorbidities. The primary aim of surgery is to completely excise the tumour with a margin of normal tissue. What constitutes an acceptable margin of normal tissue is not universally agreed but is commonly accepted as 1 cm soft tissue, or equivalent (e.g. a layer of fascia). However, on occasion, anatomical constraints mean that a true wide resection is not possible without the sacrifice of critical anatomical structures (such as major nerves, or blood vessels) and in this situation, it may be acceptable to leave a planned microscopic positive surgical margin, having considered the risks of recurrence and morbidity of more radical surgery and having discussed these fully with the patient [42]. It is recognised that there is a group of low-grade tumours, which have a low risk of local recurrence and metastasis (e.g. atypical lipomatous tumours, see “Lipomas and atypical lipomatous tumours” section), and it may be appropriate to treat these by planned marginal excision. In some situations, amputation may be the most appropriate surgical option to obtain local control and offer the best chance of cure.

For cases where a compartmentectomy or significant muscle resection to obtain clear margins will be required, reconstruction with free-functioning, or pedicled, muscle transfer may be considered at the time of primary surgery. This has the advantage of a single operative episode for the patient, but risks performing a definitive reconstructive procedure before clear margins have been histologically confirmed.

For patients who have undergone surgery and have an unplanned positive margin, re-excision should be undertaken if adequate margins can be achieved with acceptable morbidity. Macroscopic residual disease imparts a poor prognosis and local control is unlikely to be achieved even with addition of post-operative radiotherapy [43].

Patients with tumours that, because of size or position, are considered borderline resectable should be considered for neo-adjuvant treatment with chemotherapy (systemic or regional), or radiotherapy [44]. This decision will be guided by the histology of the tumour, likely sensitivity to systemic treatment, and the performance status of the patient (see below). Pre-operative radiotherapy should always be considered for myxoid liposarcoma due to the high response rate [45]. For other subtypes however, a significant reduction in size of the tumour is less likely so the aim may instead be to devitalise the margins of an anticipated marginal excision.

#### Surgery in the presence of metastatic disease

Surgical resection of the primary tumour remains an option as a palliative procedure in patients with metastatic disease. However, radiotherapy or chemotherapy may be more appropriate and the decision must take into account factors such as the patient’s likely prognosis, symptoms (e.g. pain or ulceration), co-morbidity, the expected morbidity of surgery, histological sub-type and the extent of metastases.

#### Isolated limb perfusion

Where available, isolated limb perfusion (ILP) may be a useful pre-operative technique for reducing the size of difficult, but potentially resectable, tumours in an extremity where limb preservation may not otherwise be possible. ILP employs local high-dose chemotherapy (melphalan) plus tumour necrosis alpha (TNFα), and hyperthermia, restricted to the affected limb using arterial and venous cannulation and a tourniquet. ILP has been shown to shrink peripheral tumours, thus rendering them operable, and should be considered in selected cases [46, 47]. It is also of particular importance as an adjunct to surgical resection for local recurrence in the post-radiotherapy setting where further radiotherapy cannot be delivered and close margins are likely. In addition, ILP may be considered for palliation of unresectable sarcomas that would otherwise require an amputation, although if the tumour subsequently remains inoperable the durability of response is limited. Currently there is only limited availability of this service for STS, at the Royal Marsden Hospital in London, and the Beatson Cancer Centre in Glasgow.

### Radiotherapy

#### Adjuvant radiotherapy

Both pre- and post-operative radiotherapy are considered to be standard approaches for most intermediate or high-grade soft tissue sarcomas. The addition of radiotherapy to surgery allows preservation of function with similar local control rates, and survival, to radical resection (i.e. compartmental excision/amputation) [48]. The majority of patients with low-grade tumours will not require radiotherapy. However, it should be considered for those with large, deep tumours with close or incomplete margins of excision, in whom re-excision is not possible, especially if adjacent to vital structures that could limit further surgery in the future. Patients who have undergone a compartmental resection or amputation do not require adjuvant radiotherapy assuming that the margins are clear.

The recommended post-operative radiation dose is 60–66 in 1.8–2 Gy fractions [49]. A two-phase technique using a shrinking field is commonly employed for limb sarcomas; 50 Gy to the initial larger volume followed by 10–16 Gy to a smaller volume [49]. This dose may need to be reduced if the field includes critical structures (for example the brachial plexus). Intensity-modulated radiation therapy (IMRT) should be considered to optimise the treatment volume, improve dose conformity, and reduce toxicity [50]. A phase II study of IMRT for sarcoma is underway in the UK, and ideally patients should be treated within the trial setting (IMRiS. Health Research Authority) [51].

The VORTEX clinical trial of post-operative radiotherapy for extremity soft-tissue sarcomas has completed recruiting in the UK [52]. This randomised clinical trial is comparing the standard post-operative two-phase, shrinking field, radiotherapy technique, with a single phase applied to a smaller treatment volume. The aim is to potentially spare normal tissue, and hence improve subsequent limb function, without compromising local control. The preliminary results of the study should be available in 2016 and may influence standard practice.

Pre-operative radiotherapy in limb sarcoma utilises a lower dose of 50 Gy as well as a smaller treatment volume covering the pre-operative tumour volume rather than the post-operative tumour bed. It has been shown to be associated with increased acute, post-operative complications compared to the standard post-operative treatment, but less late toxicity, with equivalent tumour control [53, 54]. In the UK, pre-operative radiotherapy has become routine in some centres. It may be preferred particularly where the size of the radiation field required for post-operative treatment is likely to be associated with significant late morbidity, or when the tumour is of borderline operability and pre-operative radiotherapy might render the tumour operable [44], or devitalise the margins of an anticipated marginal excision. If pre-operative radiotherapy is used there is a slightly higher incidence of post-operative morbidity including acute wound healing problems. Approaches which include the use of local or free flaps might be advantageous to avoid wound complications. Free flaps may reduce the risk of post-operative wound breakdown, minimise the dead space, and reconstruct the defect. A two team surgical approach (resection and reconstruction) reduces the operative time. Pre-operative radiotherapy may be less appropriate in cases where wound healing is more likely to be problematic, such as proximal thigh/groin or axillary locations. In addition, if a patient has a rapidly growing, painful tumour early surgery may be preferred. For certain radiosensitive histological subtypes, such as myxoid liposarcoma, pre-operative radiotherapy may be particularly advantageous, given the degree of tumour shrinkage that can be achieved [44, 45]. The standard regimen for pre-operative radiotherapy is 50 Gy, over 5 weeks, followed by surgery approximately 4–6 weeks after completion of radiotherapy. A further 10–16 Gy may be given post-operatively if tumour margins are positive, after careful consideration, although recent evidence suggests this is unlikely to be beneficial and may result in excess late toxicity [55].

#### Definitive radiotherapy

The use of radiotherapy alone is unusual in the treatment of sarcoma. However, in a small number of cases the sarcoma may be considered unresectable due to location, local invasion, or because resection would lead to unacceptable morbidity or a poor functional outcome. In these cases, radiotherapy can occasionally provide a durable remission although local recurrence rates are high. Outcomes appear related to tumour size, grade, and radiation dose [56–59]; doses of over 60 Gy may be employed. In patients with significant life-limiting comorbidities lower dose, palliative radiotherapy is an option.

#### Proton therapy

Proton therapy is a highly specialised method of delivering high-dose radiotherapy to a target volume, whilst minimising dose to surrounding normal tissue. It is considered for a number of defined indications which may include spinal or paraspinal soft-tissue sarcomas in both adults and children [60]. It is commissioned by NHS England where applications for treatment are considered by a “Proton Panel”. Currently patients are sent overseas for treatment, but two new facilities are under construction at The Christie NHS Foundation Trust in Manchester and University College Hospital (UCLH) NHS Foundation Trust. The services in these centres are due to commence in 2018 and 2019 [61].

### Chemotherapy

#### Adjuvant chemotherapy

The role of adjuvant chemotherapy for most STS remains unproven. Although currently not regarded as standard treatment in the UK, there is conflicting evidence, and it may be considered for individual patients with higher risk tumours and potentially chemo-sensitive subtypes on the basis that benefit cannot be excluded. Table 3 provides a general guide as to likely relative chemosensitivity. Due to a lack of published comparative data the table is based on the referenced paper [62], modified in light of the clinical experience of the authors and reviewers of these guidelines. In most cases treatment of relapsed disease is palliative and the best chance of obtaining cure is therefore with primary treatment. In those subtypes with particularly poor prognosis, such as cardiac sarcoma, where salvage treatment for relapse would be difficult, the threshold for using adjuvant chemotherapy may be lower. It may also be considered in other situations where local relapse would be untreatable or where adequate radiotherapy could not be administered owing to the sensitivity of adjacent structures, for example spinal cord. A meta-analysis published in 1997 reported an improvement in local control and progression free survival; however, although there was a trend towards an overall survival benefit this was not statistically significant [63]. These data have been supported by two more recent overviews [64, 65]. The latter did not use original trial data and included a large Italian trial which, when published in 2001, reported a significant survival benefit for adjuvant chemotherapy; although this has not been maintained with long-term follow-up [66]. The final data from EORTC 62,931 [67], the largest trial of adjuvant chemotherapy for STS, have failed to demonstrate any clear benefit from chemotherapy in local control, relapse-free survival or overall survival in patients treated with adjuvant chemotherapy. Interestingly however, it did demonstrate improved survival in both study arms compared with previous trials, perhaps due to improved surgical techniques and increased use of adjuvant radiotherapy.

**Table 3:** Soft tissue sarcomas grouped by chemosensitivity

Relative chemosensitivity	Examples of soft tissue sarcomas
Chemotherapy integral to management	Ewing’s sarcoma family tumours
	Embryonal and alveolar rhabdomyosarcoma
Chemosensitive	Desmoplastic small round cell tumour
	Synovial sarcoma
	Myxoid/round cell liposarcoma
	Uterine leiomyosarcoma
Moderately chemosensitive	Pleomorphic liposarcoma
	Epithelioid sarcoma
	Pleomorphic rhabdomyosarcoma
	Leiomyosarcoma
	Angiosarcoma
Relatively chemo-insensitive	Malignant peripheral nerve sheath tumour
	Myxofibrosarcoma
	Dedifferentiated liposarcoma
	Clear cell sarcoma
	Endometrial stromal sarcoma
Chemoinsensitive	Alveolar soft part sarcoma
	Extraskeletal myxoid chondrosarcoma

Modified from R. Salgado and E. van Marck [62] by a consensus view of the authors and other guideline contributors

One of the issues with trials of adjuvant treatment up until now is the blanket approach of a standard chemotherapy combination for all sarcomas. It is hoped that as more effective treatments are developed for specific sarcoma subtypes, these could be tested in the adjuvant setting with a greater chance of benefit.

#### Neo-adjuvant chemotherapy

The data to support neo-adjuvant chemotherapy for STS is mainly limited to retrospective series, and phase 2 trials [68]. However pre-operative chemotherapy, or chemoradiotherapy, may be considered for those patients with large high-grade tumours that are considered borderline resectable by the sarcoma MDT [69]. The age, and any comorbidity of the patient, together with the histology of the tumour need to be taken into account. There is a wide variation in chemosensitivity between different histological subtypes (Table 3). If the tumour is chemosensitive and adjacent to critical organs, then chemotherapy may potentially render the tumour suitable for conservative surgery whereas otherwise more radical surgery would be necessary. For example, for synovial sarcoma response rates of 28% [70] in a recent review of European trials, to over 50% in a single-centre series, have been reported [71, 72]. Similarly, myxoid liposarcomas are considered to be significantly more responsive than the majority of STS [72, 73], although radiotherapy alone may be sufficient [44]. With variable response of individual tumours to chemotherapy, the tumour should be monitored closely due to the risk of progression on treatment, in which case surgery can be expedited.

### Key recommendations

1. Surgery is the standard treatment for most patients with localised STS.
2. For those patients with resectable disease, a wide excision through normal uninvolved tissues is the surgical procedure of choice.
3. Defining a “wide” margin is controversial, but with the addition of effective adjuvant therapy (e.g. radiotherapy) a tumour free margin (R0) may be adequate.
4. Where a wide excision is not possible due to anatomical constraints, a planned marginal or microscopically positive margin against a critical structure, plus radiotherapy, for intermediate and high grade tumours, may be an appropriate means of achieving tumour control while maintaining physical function.
5. Occasionally, amputation should be undertaken as the only surgical option to achieve adequate margins.
6. For patients with borderline resectable tumours, pre-operative treatment with chemotherapy and/or radiotherapy should be considered depending on histology.
7. Pre- or post-operative radiotherapy is recommended along with surgical resection of the primary tumour for the majority of patients with high-grade tumours, and for selected patients with large or marginally excised, low-grade tumours.
8. The recommended dose for post-operative radiotherapy is 60–66 Gy.
9. Pre-operative radiotherapy is advantageous in terms of better long-term functional outcome, with equivalent rates of disease control, when compared with post-operative radiotherapy. There is however an increased risk of acute post-operative wound complications.
10. The recommended dose for pre-operative radiotherapy is 50 Gy.
11. Adjuvant chemotherapy is not routinely recommended but could be considered in situations where achieving local control is likely to be compromised, or the risk of metastatic disease is particularly high, with a lower threshold for its use in the more chemosensitive sarcoma subtypes.

## Prognosis and follow-up for primary disease

Prognosis following primary treatment can be estimated by well-established nomograms based on grade, depth, size, and diagnosis as well as patient age [74]; some specialist centres have made online calculators available [75]. It appears that outcomes may have improved over the past 20 years [76], although NCIN data are less convincing [12]. Local recurrence is related to grade, margins of excision, and use of radiotherapy. Whilst most events will arise in the first 5 years following diagnosis late relapses may occur, according to this French Sarcoma Group study, particularly in retroperitoneal or very large STS [77].

In common with other tumour sites, there are few published data supporting specific follow-up protocols for STS patients, and there is an urgent need for research. Patients may be reassured by follow-up, and early detection of local relapse or pulmonary metastases may improve prognosis in some patients. Follow-up should be discussed with the patient and the rationale and limitations explained.

A survey on follow-up illustrated how varied the approach is at different centres, with no agreement on imaging, follow-up intervals, or total duration of follow-up [78]. Practices such as discharging patients treated for low-grade tumours at 5 years, when evidence suggests they recur late, require review. A recently reported trial comparing standard follow-up, with greater intensity follow-up and more imaging, failed to show any difference in outcome [79]. Furthermore, a recent retrospective study of follow-up for detection of local recurrence, demonstrated that most are detected clinically, casting doubt on the utility of routine surveillance MRI scanning [80].

It is recommended that standard follow-up consists of:

1. Clinical history,
2. Clinical examination to focus on local recurrence, with imaging using ultrasound or MRI where indicated by clinical suspicion, or if the primary site is difficult to examine clinically (e.g. pelvic tumours),
3. Chest X-ray, with subsequent CT used for investigating abnormalities.
4. Monitoring for late-effects of treatment.

In certain cases, this standard follow-up can be extended or adapted according to individual risk or local practice. If a patient were deemed unfit either for pulmonary metastasectomy or systemic treatment, then diagnosing metastases when the patient is asymptomatic has little purpose, so for example, the chest X-ray could be dispensed with; indeed, referral back to primary care might be most appropriate.

As per the ESMO guidelines [28], it is recommended that patients with intermediate/high grade tumours, which most commonly relapse within 2–3 years, should be followed every 3–4 months in the first 2–3 years, then twice a year up to the fifth year, and once a year thereafter for a minimum of 8–10 years. It is recommended that patients with low-grade tumours should be followed up every 4–6 months for 3–5 years, then annually thereafter, for at least 10 years. In low-grade sarcoma where the risk of local recurrence is the main reason for follow-up, suitably educated patients, with tumours resected from easily examined regions can be considered for discharge from formal follow-up, with an option to self-refer back to the service if any abnormality is identified.

A further value of follow-up is to monitor for adverse, late effects of treatment. Patients who have received radiotherapy may, for example be at risk of second malignancies or accelerated atherosclerosis in the radiotherapy field. Following chemotherapy there may be deterioration of renal function, and reduced fertility. In women, early menopause may require interventions for issues such as bone health. Investigations for late-effects of treatment should be considered such as full blood count, renal profile, hormone profile, and echocardiography. Patients treated in childhood for paediatric sarcomas may be handed on to adult services, and it is important that suitable follow-up continues. Survivorship is an area of cancer management on which there has been more focus in recent times. Physical disability is a major feature of the survivorship experience of patients treated for soft tissue sarcoma [81], and follow-up should support the patient in trying to minimise the impact of their treatment. Low activity levels put sarcoma survivors at further cardiovascular risk, which should be considered when constructing a follow-up regimen.

### Key recommendations

1. It is recommended that patients with intermediate or high-grade sarcoma are followed up every 3–4 months for the first 2–3 years, then twice a year for up to 5 years, and annually thereafter for a total of 8–10 years.
2. Patients with low-grade sarcoma should be followed up every 4–6 months for 3–5 years, then annually.
3. Standard follow-up practice should consist of:
  - Review of any new symptoms reported by the patient,
  - Clinical examination to focus on local recurrence, with imaging follow-up where indicated by clinical suspicion,
  - Routine chest X-ray to exclude pulmonary metastases,
  - Monitoring for late-effects of treatment.
4. New models of follow-up warrant further investigation.

## Prognosis and treatment of advanced disease

In almost all cases, the treatment intention for metastatic disease is palliation. Approximately 50% of patients with high-grade sarcoma develop distant metastases and eventually die of disseminated disease, with a median survival of approximately 12 months from diagnosis of metastases [82–84]. There are more recent data suggesting that this survival figure may be rather conservative with some improvement in outcomes over time to a median of around 18 months [85, 86].

The management of advanced disease is complex; the approach to palliative treatment depends to some extent on whether or not symptoms are present, and the potential toxicities of treatment. In order to achieve control of symptoms such as pain, or dyspnoea, it is often necessary to achieve some degree of tumour shrinkage. Clearly however in the absence of significant symptoms, disease stabilisation is an equally valid aim, to prolong good quality of life. A consistent finding in studies of soft tissue sarcoma is that overall survival, as in GIST [87, 88], is defined by absence of disease progression, not degree of response.

The treatment of advanced disease may involve a combination of various strategies, often used in a stepwise fashion, particularly for those patients with a prolonged disease course. The options will take into account the disease histology, distribution, volume, plus likely sensitivity to systemic treatment. Along with systemic treatment, surgery and radiotherapy may be considered to target symptomatic metastases or in an attempt to prolong the remission period. Other techniques, such as microwave or radiofrequency ablation, may have a role. Medications for pain or other complications such as bone metastases may be considered. Bisphosphonates or denosumab may be useful in reducing fracture risk or bone pain, based on data from other cancers, although radiotherapy or surgery may also be indicated. In some patients, metastases may behave fairly indolently and periods without active treatment are often appropriate. Other areas to focus on are good supportive care, potentially involving specialist palliative care services, in coordination with primary care.

For a number of patients, particularly those with poor performance status or significant comorbidities, standard supportive care with symptom control alone, is often the most appropriate option. Early involvement of community palliative care teams should be considered in all patients with advanced disease.

### Systemic anti-cancer therapy (SACT) for sarcoma

The development of optimal treatment protocols is hampered by the rarity and heterogeneity of sarcoma. The incidence of many of the individual sub-types of soft tissue sarcoma is too small to permit large-scale prospective randomised controlled trials. Accordingly, data are gathered from a range of studies which include single-site and multisite phase 2 trials, retrospective case series, sub-analyses of trials for which a range of histological subtypes are included and, for the rarer sub-types, individual case reports.

A national algorithm produced by the NHS England Sarcoma Clinical Reference Group (CRG) has been proposed to guide the systemic treatment of sarcoma in England; a draft is under review. This is likely to be endorsed by the British Sarcoma Group and should therefore be used alongside this guideline.

The published response rates for chemotherapy in STS vary enormously, from 10 to 50% depending on the drugs used, patient selection, and histological subtype (Table 3). It has been established that good performance status, young age, and absence of liver metastases predict a good response to chemotherapy and improved survival time [83]. It is increasingly understood that response rate is only one measure of treatment efficacy with many of the newer therapies leading to a clinical benefit through disease stabilisation. A differential response to chemotherapy according to histological subtype has been noted, and as knowledge increases it is expected that it will become increasingly possible to individualise treatment. For example; synovial sarcoma, leiomyosarcoma and myxoid liposarcoma are recognised as having higher response rates to chemotherapy. Conversely, alveolar soft part sarcoma, extraskeletal myxoid chondrosarcoma and solitary fibrous tumour are generally regarded as insensitive to chemotherapy, and there are only occasional reports of responses in clear cell sarcoma. However, in the era of targeted therapies merely looking at response rates to standard chemotherapy is starting to be superseded by more specific relationships between histology and therapeutics.

Current and future trials are focusing on targeting new therapies more specifically, utilising genomic profiling, a better understanding of tumourogenesis, and the mechanisms of drug activity. In addition, a better understanding of the immune system has led to the development of new agents such as the immune-checkpoint inhibitors which are showing great promise in other tumour types. A significant challenge in sarcoma management is that given the rarity of the disease, and the numerous subtypes, it is difficult to perform large enough trials to gain the gold-standard, randomised evidence, that is preferred when developing treatment recommendations. It therefore means that, in contrast to other cancers, treatment may be given on the basis of phase II trials, small randomised phase III trials, and even for the very rare subtypes, case series. It is important therefore, where possible to develop multicentre clinical trials and recruit patients into them. Increasingly it is clear that rather than treating sarcoma as one condition, systemic treatment should be tailored to the histology or genetics of the individual subtype [89–91].

#### Selection of SACT

As noted above, in the UK the proposed national SACT Algorithm should guide systemic treatment of sarcoma. In addition, treatment of advanced disease may involve other modalities such as radiotherapy or surgery, and so multidisciplinary team review is important. Systemic treatment should ideally be guided by established protocols, preferably shared nationally. There is potential variability in dosing and administration, particularly in the use of agents such as ifosfamide, and care should be taken to use treatment protocols that maximise benefit whilst ensuring optimal management, and minimisation, of potential toxicities. Techniques such as the use of ambulatory infusions can be used to enhance patient convenience, and free-up valuable inpatient resources [92].

In a cost-constrained health system there is a challenge to fund all active agents, particularly in rare diseases. Some of the treatments considered may fall outside current standard NHS funding, and this will have to be taken into account when discussing the options with patients.

In most cases of metastatic soft-tissue sarcoma the choice of first-line chemotherapy will be between single-agent doxorubicin, or combined doxorubicin and ifosfamide. The latter combination has not been shown to improve survival, although delivers a higher response rate at a cost of increased toxicity [84, 93]. This may be an important consideration if the patient is symptomatic due to tumour size, or a reduction in tumour volume might facilitate other treatment options. The performance status of the patient and comorbidities will play an important role in treatment selection particularly in view of potential cardiac toxicity of doxorubicin and renal toxicity seen with ifosfamide. Treatment dose is also a consideration with higher doses shown to potentially improve efficacy [94].

Standard second-line treatment is ifosfamide, which is also used first-line where anthracyclines are contraindicated, for example in patients at high risk of cardiac complications, or patients pre-treated with anthracyclines. Clinical trials have indicated a dose–response relationship, and a dose of 9–10 g/m^2^ is recommended [95]. In unselected sarcomas the response rate is in the region of 8%, although higher response rates have been observed with high-dose (>12 g/m^2^) and continuous infusion ifosfamide regimens [28, 96, 97]. Responses may be higher in certain subtypes such as synovial sarcoma, whilst leiomyosarcoma is arguably less responsive and alternative agents may be more appropriate [98]. Ifosfamide is usually given over two to three days as an inpatient, but more recently infusional regimens administering treatment via a pump over two weeks have been utilised [99]. Treatment given in this way is usually better tolerated, but so far is most established in retroperitoneal liposarcoma, and is not yet a standard of care. Renal toxicity of ifosfamide can be significant and close monitoring is required. More rarely neurotoxicity is seen; more often in debilitated patients with low albumin levels.

An alternative second-line option is the combination of gemcitabine and docetaxel. The evidence for gemcitabine and docetaxel is greatest for uterine leiomyosarcoma. However, subsequent studies have demonstrated activity in soft tissue leiomyosarcoma and other tumour types including undifferentiated pleomorphic sarcoma [100, 101]. The GeDDiS trial in which this regimen was compared with doxorubicin in the first-line setting for all sarcoma subtypes, showed it to be non-inferior, but more toxic [102].

Trabectedin, licensed as second-line treatment for all soft tissue sarcomas, was approved by the European Medicines Agency (EMA) on the basis of a randomised trial comparing two different treatment regimens in patients with predominantly leiomyosarcoma and liposarcoma [103]. A recently completed trial in patients with leiomyosarcoma and liposarcoma comparing trabectedin with dacarbazine demonstrated significant superiority for trabectedin resulting in the drug being licensed in the USA [104]. Other tumours, such as synovial sarcoma, and particularly myxoid liposarcoma, may also be sensitive. It appears to be active in sarcomas related to chromosomal translocations [105, 106]. When assessing clinical benefit, it should be noted that a period of disease stabilisation may often occur for some time before response is seen. Trabectedin is currently approved by NICE and treatment can continue until disease progression. It exhibits less haematological toxicity than doxorubicin or ifosfamide but prescribers need to be aware of rare, but potentially serious rhabdomyolysis, and hepatic toxicity.

Beyond doxorubicin, ifosfamide, gemcitabine/docetaxel, and trabectedin, there are no standard chemotherapy options and decisions will be made based on patient fitness, and a balance of likely benefit and toxicities. Consideration of previous clinical benefit from chemotherapy, and more chemo-sensitive subtypes of sarcoma may support further treatment. Below are a number of options included in the proposed Sarcoma Chemotherapy Algorithm:

- Liposomal doxorubicin (Caelyx): could be considered at any line for vascular intimal sarcomas, angiosarcomas [107], cardiac sarcomas, and patients who have received previous anthracyclines, or have impaired cardiac function [108]. It can be combined with ifosfamide. It also has activity in fibromatosis (see “Desmoid-type fibromatosis” section).
- Paclitaxel: may be used as first or second line treatment of angiosarcomas [109].
- Oral cyclophosphamide and prednisolone: a low toxicity combination suitable for elderly patients unlikely to tolerate more toxic chemotherapy [110].
- Pazopanib: has data supporting its use in metastatic STS (not liposarcoma). A placebo controlled study demonstrated a 3-month improvement in progression-free survival in STS, with no particular superiority in any individual subtype. [111, 112]. Of note, activity was also seen in refractory desmoplastic small round cell tumour. This class of VEGFR inhibitor (including sunitinib) has also demonstrated activity in haemangiopericytoma/malignant solitary fibrous tumour [113], which is relatively resistant to chemotherapy (although see dacarbazine below), and in refractory desmoid tumours/fibromatosis [114].
- Dacarbazine: in the past used more commonly in STS, it has come to be used primarily for leiomyosarcoma, either as a single agent or in combination with gemcitabine [115]. Activity has also been reported against solitary fibrous tumour/haemangiopericytoma [116].

It should be noted that not all active agents mentioned above are currently funded by the NHS in the UK for the indications described. Funding varies across the devolved nations and is regularly under review.

Although not yet appraised by NICE, or commonly used in the UK, in April 2016 eribulin received marketing authorisation from the European Medicines Agency (EMA) for the treatment of unresectable liposarcoma following prior anthracycline-containing therapy. This followed subgroup analysis of a study comparing eribulin with dacarbazine for previously treated patients with liposarcoma or leiomyosarcoma [117].

#### Management of local recurrence

Local recurrences are often accompanied by metastatic disease and patients should be carefully staged for this. In the absence of overt metastatic disease every attempt should be made to regain local control by further surgery with adequate margins (wide or radical), and radiotherapy (if not used previously). Amputation may be needed in selected cases.

#### Management of lung metastases

Following a diagnosis of lung metastases, the decision regarding metastasectomy should be based on disease-free period following primary surgery, absence of other metastases, number of lesions per lung, tumour growth, and evolution of disease (ESMO 2014) [28]. In the absence of a significant disease-free interval, the CT scan (or PET-CT scan to complete staging) should be repeated at a three-month interval, and if no new lesions have appeared and the disease is operable, surgery is usually recommended. The practice of performing an interval scan and delaying surgery can be difficult to explain to patients, but the risk of immediate surgery is that further multiple metastases appear rapidly, rendering the morbidity of surgery pointless, and potentially delaying systemic treatment. Other approaches can also be considered such as radiofrequency or microwave ablation. More recently stereotactic ablative radiotherapy (SABR), a very targeted form of high-dose hypo-fractionated radiotherapy, has become another potential option. While there are few data from prospective studies reporting survival of STS patients surgically treated for thoracic metastases, there are many long-term survivors (reported variously at 20–40% of all patients undergoing lung surgery) who have had the procedure [118]. It however remains unproven that metasectomy improves long term survival.

#### Management of extrapulmonary oligometastases

In most cases extrapulmonary metastases will be treated with systemic treatment. In selected cases surgery, radiofrequency ablation (RFA), cryotherapy, or radiotherapy may be considered for limited metastatic disease to prolong remission or reduce symptoms. Electrochemotherapy (ECT) is an emerging technique that may be useful in the management of refractory dermal and subcutaneous metastases in certain tumour sub-types, for example angiosarcoma [119, 120].

#### Best Supportive Care

Supportive and palliative care should always be considered in cases of advanced disease. For many patients, systemic therapy, radiotherapy, or surgery may not be appropriate, and an early and honest conversation about treatment options, potential toxicities and quality of life is important. Involvement of a sarcoma specialist nurse to support the patient through the diagnostic process and discussion of options can be invaluable. Early referral to specialist palliative care services in the community should be considered. Although prognostication can be difficult and inexact, most patients and their families will want some idea of likely outcomes and this should be explored with them. Discussions concerning end-of-life care preferences may also be appropriate.

### Key recommendations

1. Systemic treatments for the majority of advanced STS are not curative; median survival time is 12–18 months. Published chemotherapy response rates vary enormously; from 10–50% depending on the drugs used, patient selection, and tumour grade and histological subtype.
2. Treatment recommendations should be guided by patient performance status, disease extent, rate of progression, and potential sensitivity to treatment.
3. Standard first-line treatment is single-agent doxorubicin.
4. Ifosfamide may be used first-line if anthracyclines are contraindicated, and is a standard option for second-line therapy.
5. Although the combination of doxorubicin and ifosfamide has not been demonstrated to improve survival in comparison to single agent doxorubicin first-line, response rates are higher and it may be considered in individual patients where a response would improve symptoms or facilitate other treatment modalities.
6. Additional second-line agents include trabectedin, and the combination of gemcitabine and docetaxel. The choice of agent depends on histology, toxicity profile and patient preference.
7. A number of other agents such as dacarbazine and pazopanib can be considered beyond second-line depending on patient fitness and funding constraints.
8. Surgical resection of locally recurrent disease should be considered where feasible. For patients with oligometastatic disease surgery, radiotherapy, or ablative therapies (RFA, SABR, cryotherapy, microwave, ECT) should be considered in individual cases, although there are limited data on survival benefit.

## Uterine and retroperitoneal sarcomas

Given the heterogeneity of sarcoma presentations many patients are managed in collaboration with other multidisciplinary teams. For England, reference should be made to the NICE Quality Standard, QS78 [41] and National Sarcoma Service Specification [8]. The MDTs should combine expertise to ensure optimal management taking into consideration tumour location, and subtype. Uterine sarcomas in the UK are usually managed primarily by regional gynaecological cancer MDTs, but strong links to the sarcoma MDT should be maintained to ensure that patients are appropriately registered, managed, considered for clinical trials, and referred for systemic treatment if required for metastatic disease. Retroperitoneal sarcomas should be managed by surgeons who are members of the sarcoma MDT although not every UK STS centre will have this service available and cross-referral may be required. Gastrointestinal stromal tumours (GIST) are usually managed in collaboration with GI surgical services, and are discussed in separate BSG guidance.

### Uterine sarcomas

This group includes uterine leiomyosarcomas (LMS), endometrial stromal sarcomas (ESS), and undifferentiated endometrial sarcoma (UES). Standard treatment for all localised tumours is total abdominal hysterectomy (TAH), with some differences between the tumour types as described below [28]. Carcinosarcomas (malignant mixed Mullerian tumours, MMMT) are considered as epithelial tumours and, although new biological insights are emerging [25], should be treated accordingly, unless the sarcomatous element predominates.

#### Uterine leiomyosarcoma

Uterine LMS, a cancer of the smooth muscle, accounts for 35–40% of all uterine sarcomas; LMS can affect young women in their mid-20 s, although most patients will be aged 50–60 years. Pre-operatively it is difficult to differentiate benign leiomyomas from malignant LMS and so the surgical approach should be planned accordingly; laparoscopic morcellation is contraindicated for uterine sarcoma due to higher risk of recurrence and metastasis [121–123]. The risk of inadvertent morcellation of a uterine sarcoma increases significantly with age [124], and the US Food and Drug Administration (FDA) released a safety communication concerning the procedure in 2014 [125]. Standard surgical management for non-metastatic disease is total abdominal hysterectomy (TAH) with, or without bilateral salpingo-oophorectomy (BSO). Retention of the ovaries can be considered in pre-menopausal women. Lymphadenectomy is not routinely required as incidence of lymph node involvement is less than 5%. Adjuvant pelvic radiotherapy for FIGO stage I and II disease is not recommended routinely [126]. Adjuvant pelvic radiotherapy may be considered for selected high-risk cases, for example after tumour rupture, where local relapse may be reduced, although a survival benefit has not been demonstrated [127]. Adjuvant chemotherapy is not routinely recommended but, as for other STS, can be considered in high-risk disease where there is some limited evidence [128, 129]. Chemotherapy for advanced/metastatic disease is as for STS at other sites, with doxorubicin as first line, although ifosfamide may be relatively less effective in LMS [98]. The combination of gemcitabine and docetaxel has demonstrated activity in the second-line setting in leiomyosarcoma. Trabectedin also seems to have useful activity [130].

Oestrogen receptor (ER) and progesterone receptor (PgR) expression is seen in approximately 50% of patients with uterine LMS. Some low and intermediate grade tumours may be sensitive to oestrogen deprivation, e.g. using aromatase inhibitors, although there are very few published data on this situation [131]. It is however reasonable to look for receptor expression in those with relatively indolent tumours for which treatment with an aromatase inhibitor or a progestogen might be appropriate. However, receptor expression does not guarantee response to oestrogen-lowering therapy, and use of oestrogen-lowering therapies should be used with particular caution in patients with high-grade rapidly progressing tumours.

#### Endometrial stromal sarcoma

Although a rare uterine malignancy, this is the second most prevalent uterine sarcoma, and a generally indolent disease with a long natural history. It was formally known as “low grade ESS”, on the basis of a mitotic count of less than 10 mitoses per 10 high powered fields, but is now termed simply ESS, with no distinction between “grade” (mitotic count is now recognised not to be prognostic). There is a high incidence of oestrogen (ER) and progesterone receptor (PR) expression, and evidence that these tumours are hormonally responsive. Standard surgical treatment is therefore total abdominal hysterectomy, with bilateral salpingo-oophorectomy in pre-menopausal women; hormone replacement therapy (HRT) is contraindicated postoperatively [132]. A single small study has suggested that adjuvant progestogens after surgery may improve outcome; routine use is not indicated but could be considered in high risk patients [133]. The role of adjuvant pelvic radiotherapy is uncertain given the paucity of published data. Recurrent or metastatic disease may respond to anti-oestrogen therapy, with an aromatase inhibitor, or a progestogen. Tamoxifen is not recommended since its action may be pro-oestrogenic in this setting. Chemotherapy is an option if hormonal therapy fails. Given the indolent nature of the condition, surgery for metastatic disease should be considered.

#### Undifferentiated endometrial sarcoma

This disease entity was formally known as “high grade ESS”, but is now termed undifferentiated endometrial sarcoma (UES). It is a highly aggressive anaplastic malignancy that does not express ER and PR, with a poor prognosis even for early stage disease, and uncertain response to systemic treatment. Surgical management is TAH with or without BSO, and the option of adjuvant pelvic radiotherapy [126]. Follow-up protocols and systemic treatment for advanced disease parallel those for adult-type soft tissue sarcomas [28]. Oestrogen-lowering therapies are generally not used.

There has been some reported success with cisplatin in treating uterine sarcomas but figures are distorted because of high numbers of carcinosarcoma/malignant mixed Mullerian tumour (MMMT) patients in the only large trial. No subset analysis has been offered, therefore this drug is not recommended.

#### Key recommendations

1. Standard treatment for all localised uterine sarcomas is TAH. Lymphadenectomy is not routinely indicated.
2. Total abdominal hysterectomy, with bilateral oophorectomy is indicated for endometrial stromal sarcoma. These patients should not have post-operative hormone replacement therapy, and tamoxifen is contraindicated. Use of adjuvant oestrogen deprivation therapy is not routinely indicated.
3. Adjuvant pelvic radiotherapy has not been shown to improve survival, and is not routinely indicated in FIGO stage I and II disease. However, it could be considered for selected high-risk cases.
4. Advanced/metastatic LMS and undifferentiated endometrial sarcoma are treated systemically with the same drugs as STS at other sites. There is retrospective evidence that ifosfamide may be less effective in leiomyosarcoma.
5. Advanced/metastatic ESS can be treated with oestrogen deprivation therapy, with an aromatase inhibitor or progestogen.

### Retroperitoneal sarcomas

Although the principles of management of retroperitoneal sarcomas are similar to those for extremity tumours, there are some important differences. Surgical management should be by surgeons specialised in the management of retroperitoneal sarcoma who are members of the sarcoma MDT.

Contrast-enhanced CT of the chest, abdomen, and pelvis is used for staging and may be a valuable aid to diagnosis of well-differentiated/dedifferentiated liposarcoma, and in helping to plan surgery. In most cases biopsy will be required to confirm the diagnosis, although may be considered unnecessary if the radiological appearances are typical for retroperitoneal liposarcoma. The biopsy track should be planned to reduce any risk of tumour seeding or complications.

Complete primary macroscopic resection gives the best chance of long-term cure and so the importance of surgical planning is paramount. Surgical margins are often more difficult to define as transcoelomic spread with distant contamination within the abdomen may occur. The goal of ‘wide excision’ is unlikely to be achievable in most cases. Here, the objective is “planned marginal excision”, achieving appropriate margins that balance tumour control with minimising operative morbidity and retaining function. However, multi-visceral resection may be appropriate if necessary to permit “*en bloc*” resection of tumour, organs frequently sacrificed include kidney and spleen, and partial organ resection and vascular reconstructions may occasionally be required. Pre-operative assessment of contralateral renal function should be considered. Resection of tumour leaving behind gross macroscopic disease is of limited benefit and may cause unnecessary morbidity. Studies have shown the importance of adherence to proper surgical guidelines in the management of this disease, with a direct impact on survival [134].

The role of pre or post-operative radiotherapy is less well defined, and although it may be of value in individual patients, it is not considered routine. It is often difficult to define the radiation volume and dose is limited due to the risk of small bowel and other organ toxicity. In cases where it is possible to define “high-risk margins” post-operative radiotherapy to a dose of 45–50 in 1.8 Gy fractions should be considered [135]. In certain situations, for example, low pelvic tumours, higher doses of radiation may be given as normal tissue tolerance is greater. Pre-operative radiotherapy is increasingly becoming a preferred option as the treatment volume is smaller and better defined and the tumour acts as its own “spacer” [136]. Currently the STRASS trial, randomising patients with RPS to surgery or surgery plus pre-operative radiotherapy, is recruiting patients [137].

There is currently no evidence to support the use of neo-adjuvant or adjuvant chemotherapy in the management of retroperitoneal sarcomas, although as in other STS is may be considered in more sensitive histologies such as synovial sarcoma.

Routine CT scanning for asymptomatic relapse is controversial due to the relative ineffectiveness of salvage surgery. However, surgery for local recurrence may be considered in cases where there has been a reasonable disease-free interval (over 12 months) particularly in low-grade disease, or disease demonstrating a good response to systemic treatment.

Palliative chemotherapy should be considered for the same indications as limb sarcomas but well-differentiated/de-differentiated liposarcoma is relatively chemoresistant. Options usually include doxorubicin, infusional ifosfamide, or trabectedin any of which might be considered in the first-line setting. Early data suggests that eribulin may have a role [117] although it has not yet been appraised by NICE.

For many patients with advanced disease, aggressive therapy may not be appropriate, and good symptomatic management, and palliative care support are required.

#### Key recommendations

1. Standard treatment is *en bloc* complete resection with macroscopically clear margins.
2. Treatments for relapse are relatively ineffective; symptomatic management and palliative support of the patient should be offered where appropriate.

## Borderline tumours

This group of soft tissue tumours are not considered typical sarcomas. They tend to remain localised, and whilst local recurrence following surgery can occur, they do not generally metastasise.

### Lipomas and atypical lipomatous tumours

The most common differential diagnosis seen by the sarcoma MDT is that between lipoma and atypical lipomatous tumours (ALT), also known as well-differentiated liposarcoma (WDL). Essentially ALT and WDL are synonymous, as described in the WHO classification [10]. The latter term is more commonly applied to tumours in sites such as the retroperitoneum and mediastinum where surgical excision with a wide margin is unlikely, and therefore local recurrence almost inevitable; progressive dedifferentiation with each recurrence is often observed (see “Retroperitoneal sarcomas” section). ALT/WDL of the extremities is distinct from lipoma in that it has the propensity for local recurrence, however dedifferentiation into a more aggressive disease is extremely rare.

Differentiating lipoma and ALT radiologically is not reliable but certain features seen on MRI can be helpful such as size and intratumoural septation [138, 139]. Histological and cytogenetic analysis of tumour allows confirmation of diagnosis, although small pre-operative biopsies may be misleading [140, 141].

Surgical resection is the usual treatment for ALT, and the prognosis is usually excellent [142–144]. However, particularly in older patients, if surgery is likely to be morbid, or the patient has significant comorbidities then radiological surveillance can be considered. In larger tumours, or those where clear margins are difficult to achieve, adjuvant radiotherapy may very occasionally be considered [145].

#### Key recommendations

1. Atypical lipomatous tumours and well differentiated liposarcomas are essentially synonymous. Surgical resection with a clear margin is standard treatment and prognosis is usually excellent.

### Desmoid-type fibromatosis

Fibromatosis is a benign, clonal tumour, which although sometimes locally aggressive (even fatal on occasion), has not been reported to metastasise. Although usually sporadic it may occur in association with familial adenomatous polyposis (FAP), or Gardner syndrome caused by germline mutations in the *APC* gene. Cases of sporadic fibromatosis usually harbour mutations in *CTNNB1*, the gene for beta-catenin.

Diagnostic investigation follows the standard process for STS. The disease may occur at the sites of previous scars and can be related to hormonal changes in women, for example surrounding pregnancy. Pregnancy related fibromatosis tends to have a good outcome and progression during pregnancy is common but manageable. It is not generally a contraindication to future pregnancy [146]. In cases where a link to FAP may be more likely (e.g. young male, abdominal disease, *CTNNB1* mutation negative) then it is important to exclude a family history of bowel cancer, and it may be appropriate to screen for germline *APC* mutations or consider investigations such as colonoscopy.

The natural history of the disease is unpredictable and optimal management is not fully established, although a European Consensus has recently been published and provides useful guidance [147]. Following initial diagnosis, the tumour may continue to grow, stabilise, or even regress spontaneously [148]. The 5-year progression-free survival may be up to 50%. In addition, surgical resection even with apparently clear margins results in relatively high rates of local recurrence in up to half of cases. Increasingly, watchful waiting is considered the standard first-line option. Interval review with MRI scans is recommended and treatment initiated on significant disease progression.

Standard treatment in cases where surveillance is not selected, or progression has occurred, is complete surgical excision. Unlike the general situation with STS the finding of positive margins is less closely related to risk of relapse; long-term remission may be seen despite positive margins, and conversely relapse is not uncommon in clearly resected disease. The exception to this is fibromatosis arising in the abdominal wall of young females, where relapse rates following surgery are low; in one series under 10% at 5 years [149].

Radiotherapy may be effective treatment for patients with unresectable tumours or may be given as adjuvant therapy following surgery for recurrent disease, especially if further surgery would result in significant morbidity and functional deficit. A dose of 50–56 Gy is usually employed [150, 151].

Systemic treatment is recommended in selected cases with unresectable disease and is another option following progression during watchful waiting. Hormone therapies such as tamoxifen have been reported to be beneficial but, because of the unpredictable natural history of this disease, their true value remains unproven due to the lack of appropriate clinical trial data. Nonsteroidal anti-inflammatory drugs (NSAIDs) have been reported to improve the response to tamoxifen. The precise choice of NSAID is uncertain and although selective COX2 inhibitors have been used, the evidence that they are superior is lacking. NSAIDS have an impact on the beta-catenin signalling pathway.

Chemotherapy is usually reserved for patients with significant symptoms who have failed to respond to more benign interventions such as the use of NSAIDs and tamoxifen. Weekly administration of methotrexate and vinblastine or vinorelbine has reasonable activity and is generally well tolerated. More recently pegylated liposomal doxorubicin (Caelyx) has been reported to have significant activity with acceptable toxicity, and currently is considered treatment of choice by many investigators [152]. Targeted therapies such as imatinib and pazopanib have also been investigated, and both objective remissions and disease stabilisation have been reported [114, 153, 154]. Another option for limb tumours is isolated limb perfusion (ILP) with tumour necrosis factor alpha and melphalan.

#### Key recommendations

1. A diagnosis of familial adenomatous polyposis (FAP) needs to be considered in some fibromatosis cases.
2. Initial standard treatment for fibromatosis is watchful waiting.
3. Systemic treatments such as tamoxifen, NSAIDS or chemotherapy may be used definitively, or neoadjuvantly before surgery.
4. Surgery can be considered if progression occurs.
5. Radiotherapy can be used in the adjuvant setting, or for inoperable disease.

### Peripheral nerve tumours

Peripheral nerve tumours are often referred to, or managed by, sarcoma services, and include several subtypes including neurofibromas, schwannomas, and malignant peripheral nerve sheath tumours (MPNST). They may be benign or malignant, and sporadic, or in a significant minority of cases, associated with the genetic conditions neurofibromatosis type 1 or 2. The latter conditions will not be discussed in detail in this guideline but overall management is likely to be in collaboration with regional genetic medicine or specialist neurofibromatosis services.

Presentation may be as a mass, but pain, or focal neurological symptoms may also be a feature. Rapid progression of symptoms or signs may indicate a higher likelihood of malignancy [155]. Ultrasound, and in particular MRI scans can suggest the diagnosis with features such as direct continuity with a nerve or location along a typical nerve distribution [156]. PET-CT may assist in differentiating benign from malignant tumours [32, 33].

In many cases a biopsy will be appropriate to confirm the diagnosis. However, this can be painful for the patient, or rarely associated with neurological injury. Therefore, some lesions with characteristic appearances on MRI scan may be considered for excision biopsy under general anaesthetic. Expert pathological assessment will be required as nerve tumours can be diagnostically challenging [157].

Treatment is usually surgical resection, although in some cases management can be conservative with observation alone. Surveillance may be considered in asymptomatic schwannomas, or other neural tumours with no malignant features [158]. For benign tumours resection with minimisation of residual neurological deficit is the aim, and in many cases can result in improvement in peripheral nerve function [159]. Malignant peripheral nerve sheath tumours (MPNST) are aggressive tumours with a relatively poor prognosis [160]. In general management is as for malignant soft-tissue sarcomas as described earlier in this guideline.

#### Key recommendations

1. In the presence of a peripheral nerve tumour a diagnosis of neurofibromatosis should be considered.
2. Treatment is usually surgical excision although surveillance can be considered in clearly benign cases.
3. Malignant peripheral nerve sheath tumours are aggressive malignancies treated in the same way as other high-grade sarcomas.

### Dermatofibrosarcoma protuberans (DFSP)

DFSP is a rare neoplasm of the dermal layer of the skin. This is best considered a borderline malignancy that rarely metastasises but is locally aggressive, may produce significant morbidity, and occasionally proves fatal. Local recurrence following surgery is common and wide excision is essential except in situations where wide excision would result in significant morbidity or functional loss. In this instance, Mohs surgery can provide an alternative to initial wide excision and may be delivered through collaboration with a skin cancer MDT.

Radiotherapy should be considered for inoperable disease, and can result in durable remissions. Adjuvant radiotherapy may also be used if the margins are involved and re-excision is not possible [161].

Systemic treatment is appropriate in selected cases with unresectable or metastatic disease. DFSP is driven by a t(17;22) translocation that results in over-expression of platelet derived growth factor beta (PDGF*β*). Therefore, the PDGF*β* receptor may be inhibited by imatinib, which is licensed for the treatment of unresectable DFSP [162].The challenges with using targeted agents such as this for benign conditions are balancing the toxicity with benefit, the financial cost, and also defining an appropriate end-point, and optimum duration of treatment.

#### Key recommendations

1. Treatment of DFSP is wide surgical excision, Mohs surgery may be appropriate in selected cases to reduce functional loss.
2. Adjuvant radiotherapy may be considered if surgical resection is incomplete, and re-excision not possible.
3. Imatinib may provide an option for neo-adjuvant treatment in borderline resectable disease, or effective palliation for patients with unresectable DFSP.

### Atypical fibroxanthoma (AFX)

AFX is a low-grade cutaneous spindle cell tumour considered a superficial variant of malignant fibrous histiocytoma (MFH). It may be mistaken clinically or histologically for other spindle cell tumours. It is usually cured by surgical excision although local recurrence is fairly common and metastases are seen in less than 1% of cases. AFX greater than 2 cm in size and with other adverse pathological features may be regarded as pleomorphic dermal sarcomas [163]; they appear to share similar oncogene expression and mutations [164].

#### Key recommendation

1. AFX is usually cured by surgical excision, although larger tumours with adverse pathological features may be regarded as pleomorphic dermal sarcomas.

### Tenosynovial giant cell tumour (TGCT)

This family of benign neoplastic conditions presents as two forms, reclassified by the WHO in 2013 [10], as either a single nodule (localised, L-TGCT; previously GCT of tendon sheath or nodular tenosynovitis) or multiple nodules (diffuse-type, D-TGCT; previously pigmented villonodular synovitis, PVNS), generally affecting the synovium in young adults. It is usually treated by surgery alone but local relapses can occur [165]. Arthroscopic or open synovectomy may have a role in diffuse disease. In nodular intra-articular disease, the aim is complete removal, and doing this without morsellisation may be advantageous.

The role of radiotherapy is unclear but may be considered for symptomatic residual or recurrent disease when further excision is not possible. Yttrium synovectomy has been used in the adjuvant setting in diffuse intraarticular disease [166]. Due to a translocation involving the macrophage colony-stimulating factor (*M*-*CSF* or *CSF1*) gene seen in a proportion of cells, imatinib has demonstrated activity in its treatment [167]. It may be considered for a 3-month course prior to surgery in borderline operable cases, although will require approval for funding. Its use in the palliative setting can also be considered although the treatment endpoints and duration are not clear [168]. New targeted drugs are currently undergoing investigation.

#### Key recommendation

1. Tenosynovial giant cell tumour is generally treated by surgery alone, although rarely radiotherapy or imatinib may have a role.

### Inflammatory myofibroblastic tumour (IMT)

IMT is a neoplasm consisting of a spindle-cell proliferation and inflammatory infiltrate. It most commonly occurs in the lungs but can be seen in the abdomen and pelvis or maxillofacial region. Treatment is usually surgical excision although local recurrences and very rarely metastases can occur. It may respond to steroids, but in around 50% of cases rearrangements in the *ALK* locus on chromosome 2p23 have been detected. In these cases, treatment with an *ALK* targeting drug such as crizotinib may be useful [169] and is currently being investigated in the CREATE study [170].

#### Key recommendation

1. Inflammatory myofibroblastic tumour is treated with surgery, although may be responsive to steroids or crizotinib.

## Summary of key recommendations

### Clinical presentation

1. Any patient with a soft tissue mass that is increasing in size, or has a size more than 5 cm, whether or not it is painful, should either be referred for an urgent ultrasound scan, or referred directly to a sarcoma diagnostic centre.
2. If the ultrasound scan does not definitely confirm benign pathology, then the patient should be referred for further investigation on an urgent suspected cancer referral pathway.
3. Any retroperitoneal or intra-abdominal mass with imaging appearances suggestive of a soft tissue sarcoma should be referred to a specialist centre before biopsy or surgical treatment.

### Referral and assessment

1. All patients with a suspected STS should be managed by a specialist Sarcoma MDT as specified in the NICE guidance.
2. Ultrasound scan by a musculoskeletal radiologist should be considered as the first-line investigation, and may be supplemented by ultrasound-guided core biopsy.
3. Magnetic resonance imaging and core needle biopsy are recommended prior to definitive surgery.
4. Imaging of the thorax by CT scan for lung metastases should be done prior to radical treatment. Further staging may be considered depending on subtype and location of the sarcoma.

### Management of localised disease

1. Surgery is the standard treatment for most patients with localised STS.
2. For those patients with resectable disease, a wide excision through normal uninvolved tissues is the surgical procedure of choice.
3. Defining a “wide” margin is controversial, but with the addition of effective adjuvant therapy (e.g. radiotherapy) a tumour free margin (R0) may be adequate.
4. Where a wide excision is not possible due to anatomical constraints, a planned marginal or microscopically positive margin against a critical structure, plus radiotherapy, for intermediate and high grade tumours, may be an appropriate means of achieving tumour control while maintaining physical function.
5. Occasionally amputation should be undertaken as the only surgical option to achieve adequate margins.
6. For patients with borderline resectable tumours, pre-operative treatment with chemotherapy and/or radiotherapy should be considered depending on histology.
7. Pre- or post-operative radiotherapy is recommended along with surgical resection of the primary tumour for the majority of patients with high-grade tumours, and for selected patients with large or marginally excised, low-grade tumours.
8. The recommended dose for post-operative radiotherapy is 60–66 Gy.
9. Pre-operative radiotherapy is advantageous in terms of better long-term functional outcome, with equivalent rates of disease control, when compared with post-operative radiotherapy. There is however an increased risk of acute post-operative wound complications.
10. The recommended dose for pre-operative radiotherapy is 50 Gy.
11. Adjuvant chemotherapy is not routinely recommended but could be considered in situations where achieving local control is likely to be compromised, or the risk of metastatic disease is particularly high, with a lower threshold for its use in the more chemosensitive sarcoma subtypes.

### Prognosis and follow-up for primary disease

1. It is recommended that patients with intermediate or high-grade sarcoma are followed up every 3–4 months for the first 2–3 years, then twice a year for up to 5 years, and annually thereafter for a total of 8–10 years.
2. Patients with low-grade sarcoma should be followed up every 4–6 months for 3–5 years, then annually.
3. Standard follow-up practice should consist of:
  - Review of any new symptoms reported by the patient,
  - Clinical examination to focus on local recurrence, with imaging follow-up where indicated by clinical suspicion,
  - Routine chest X-ray to exclude pulmonary metastases.
  - Monitoring for late-effects of treatment.
4. New models of follow-up warrant further investigation.

### Prognosis and treatment of advanced disease

1. Systemic treatments (SACT) for the majority of advanced STS are not curative; median survival time is 12–18 months. Published chemotherapy response rates vary enormously; from 10–50% depending on the drugs used, patient selection, and tumour grade and histological subtype.
2. Treatment recommendations should be guided by patient performance status, disease extent, rate of progression, and potential sensitivity to treatment.
3. Standard first-line treatment is single-agent doxorubicin.
4. Ifosfamide may be used first-line if anthracyclines are contraindicated, and is a standard option for second-line therapy.
5. Although the combination of doxorubicin and ifosfamide has not been demonstrated to improve survival in comparison to single agent doxorubicin first-line, response rates are higher and it may be considered in individual patients where a response would improve symptoms or facilitate other treatment modalities.
6. Additional second-line agents include trabectedin, and the combination of gemcitabine and docetaxel. The choice of agent depends on histology, toxicity profile and patient preference.
7. A number of other agents such as dacarbazine and pazopanib can be considered beyond second-line depending on patient fitness and funding constraints.
8. Surgical resection of locally recurrent disease should be considered where feasible. For patients with oligometastatic disease surgery, radiotherapy, or ablative therapies (RFA, SABR, cryotherapy, microwave, ECT) should be considered in individual cases, although there are limited data on survival benefit.

### Uterine sarcomas

1. Standard treatment for all localised uterine sarcomas is TAH. Lymphadenectomy is not routinely indicated.
2. Total abdominal hysterectomy with bilateral oophorectomy is indicated for endometrial stromal sarcoma. These patients should not have post-operative hormone replacement therapy and tamoxifen is contraindicated. Use of adjuvant oestrogen deprivation therapy is not routinely indicated.
3. Adjuvant pelvic radiotherapy has not been shown to improve survival, and is not routinely indicated in FIGO stage I and II disease. However, it could be considered for selected high-risk cases.
4. Advanced/metastatic LMS and undifferentiated endometrial sarcoma are treated systemically with the same drugs as STS at other sites. There is retrospective evidence that ifosfamide may be less effective in leiomyosarcoma.
5. Advanced/metastatic ESS can be treated with oestrogen deprivation therapy, with an aromatase inhibitor or progestogen.

### Retroperitoneal sarcomas

1. Standard treatment is *en bloc* complete resection with macroscopically clear margins.
2. Treatments for relapse are relatively ineffective; symptomatic management and palliative support of the patient should be offered where appropriate.

### Lipomas and atypical lipomatous tumours

1. Atypical lipomatous tumours and well differentiated liposarcomas are essentially synonymous. Surgical resection with a clear margin is standard treatment and prognosis is usually excellent.

### Desmoid-type fibromatosis

1. A diagnosis of familial adenomatous polyposis (FAP) needs to be considered in some fibromatosis cases.
2. Initial standard treatment for fibromatosis is watchful waiting.
3. Systemic treatments such as tamoxifen, NSAIDS or chemotherapy may be used definitively, or neoadajuvantly before surgery for fibromatosis.
4. Surgery can be considered for fibromatosis if progression occurs.
5. Radiotherapy can be used in the adjuvant setting, or for inoperable fibromatosis.

### Peripheral nerve tumours

1. In the presence of a peripheral nerve tumour a diagnosis of neurofibromatosis should be considered.
2. Treatment of peripheral nerve tumours is usually surgical excision although surveillance can be considered in clearly benign cases.
3. Malignant peripheral nerve sheath tumours are aggressive malignancies treated in the same way as other high-grade sarcomas.

### Dermatofibrosarcoma protruberans (DFSP)

1. Treatment of DFSP is wide surgical excision, Mohs surgery may be appropriate in selected cases to reduce functional loss.
2. Adjuvant radiotherapy may be considered for DFSP if surgical resection is incomplete, and re-excision not possible.
3. Imatinib may provide an option for neo-adjuvant treatment in borderline resectable disease, or effective palliation for patients with unresectable DFSP.

### Atypical fibroxanthoma (AFX)

1. AFX is usually cured by surgical excision, although larger tumours with adverse pathological features may be regarded as pleomorphic dermal sarcomas.

### Tenosynovial giant cell tumour (TGCT)

1. Tenosynovial giant cell tumour is generally treated by surgery alone, although rarely radiotherapy or imatinib may have a role.

### Inflammatory myofibroblastic tumour (IMT)

1. Inflammatory myofibroblastic tumour is treated with surgery, although may be responsive to steroids or crizotinib.
